# Clinical and Biological Factors Associated with Pseudoprogression Under Immune Checkpoint Inhibitors: A Retrospective Pan-Tumor Cohort Study

**DOI:** 10.3390/cancers18183040

**Published:** 2026-09-19

**Authors:** Amélie Toulet, Valérie Seegers, Frédéric Bigot, Sylvère Guillemois, Damien Vansteene, Rémy Delva, Manon de Vries-Brilland

**Affiliations:** 1 Department of Medical Oncology, Integrated Centers of Oncology (ICO) Paul Papin, 49055 Angers, France; amelie.toulet@hotmail.fr (A.T.); frederic.bigot@ico.unicancer.fr (F.B.); remy.delva@ico.unicancer.fr (R.D.); 2 Biostatistics Department, Integrated Centers of Oncology (ICO) Paul Papin, 49055 Angers, France; valerie.seegers@ico.unicancer.fr; 3 Department of Medical Oncology, Cholet Hospital, 49300 Cholet, France; sylvere.guillemois@ch-cholet.fr; 4 Department of Medical Oncology, Integrated Centers of Oncology (ICO) Saint-Herblain, 44805 Saint-Herblain, France; damien.vansteene@ico.unicancer.fr

**Keywords:** immune checkpoint inhibitor, pseudoprogression, LDH, ECOG, metastatic cancer, factors associated with pseudoprogression

## Abstract

**Background**: Immune checkpoint inhibitors (ICIs) have transformed cancer treatment but may induce pseudoprogression (PP), an atypical response pattern that may mimic progressive disease (PD). Although iRECIST criteria were developed to characterize such atypical responses, distinguishing PP from PD when progression is first observed remains challenging. We aimed to characterize patients with clinically suspected PP and explore clinical and biological factors associated with subsequent classification as PP versus PD. **Methods**: We conducted a retrospective single-center study including patients with metastatic cancer treated with ICIs at the Institut de Cancérologie de l’Ouest (ICO) in whom PP was clinically suspected. Clinical, biological, treatment, and outcome data were collected at ICI initiation, PP suspicion, and subsequent PP/PD classification. Associations with PP versus PD were explored using univariable and multivariable logistic regression. **Results:** Among 123 patients with clinically suspected PP, 56 were subsequently classified as PP and 67 as PD. The most frequent primary tumors were lung (38%), kidney (24%), and bladder (11%) cancers. Median time from ICI initiation to PP suspicion was 79 days. ECOG performance status of 0 at ICI initiation was more frequent in the PP than in the PD group (55% vs. 32%; *p* = 0.046). At PP suspicion, median LDH levels were lower in the PP than in the PD group (189 vs. 232.5 U/L; *p* = 0.003). After multivariable adjustment, higher LDH levels at PP suspicion remained associated with PD (adjusted OR 2.78 per 100 U/L increase; 95% CI, 1.19–6.52; *p* = 0.019). **Conclusions**: In patients with clinically suspected PP, LDH may provide complementary information but cannot reliably distinguish PP from PD when considered alone. Confirmatory imaging therefore remains essential for treatment decision-making.

## Simple Summary

Immunotherapy has revolutionized the treatment of many advanced cancers. However, in some patients, tumors may temporarily appear to grow before subsequently shrinking, a phenomenon known as pseudoprogression. Because imaging cannot reliably distinguish pseudoprogression from true disease progression when progression is first observed, treatment decisions can be challenging. We retrospectively studied patients with metastatic cancer treated with immune checkpoint inhibitors in whom pseudoprogression was clinically suspected and compared those subsequently classified as pseudoprogression with those ultimately classified as true disease progression. Among the clinical and biological factors evaluated, higher lactate dehydrogenase levels at the time of pseudoprogression suspicion remained associated with subsequent classification as true disease progression after adjustment. However, lactate dehydrogenase alone could not reliably distinguish pseudoprogression from true progression. Confirmatory imaging therefore remains essential, while routinely available clinical and biological information may provide complementary support during this period of diagnostic uncertainty.

## 1. Introduction

Immune checkpoint inhibitors (ICIs) have transformed the therapeutic landscape of oncology, significantly improving clinical outcomes and survival across several advanced malignancies [1,2,3]. Advances in the understanding of cancer biology have highlighted the critical role of the tumor microenvironment and the mechanisms by which tumor cells evade immune surveillance [4].

A major breakthrough in cancer immunotherapy was the identification of immune checkpoints, particularly *cytotoxic T-lymphocyte-associated antigen 4 (CTLA-4)*, *programmed cell death protein 1 (PD-1)*, and its *ligand*, *programmed* death-ligand 1 (*PD-L1*). These inhibitory pathways regulate immune responses and can be exploited by tumor cells to evade antitumor immunity [5]. ICIs, either on their own or in combination with other systemic therapies, are now widely integrated into the management of several advanced solid tumors [6]. Despite these advances, primary and acquired resistance remain major clinical challenges and involve complex tumor-intrinsic and immune-mediated mechanisms, including impaired antigen presentation, T-cell dysfunction and exhaustion, an immunosuppressive tumor microenvironment, and regulatory mechanisms involving non-coding RNAs such as microRNAs [7,8].

In addition to primary and acquired resistance, ICI treatment may be associated with atypical patterns of radiological response, among which pseudoprogression (PP) is one of the most challenging. First described with ipilimumab in melanoma, PP may result, at least in part, from treatment-induced immune-cell infiltration and inflammatory changes within tumor lesions [9,10,11]. It is characterized by initial radiological progression followed by subsequent tumor stabilization or response without a change in systemic anticancer treatment. Its reported incidence ranges from 0% to 15%, depending on tumor type and study population, and it has been most frequently described in melanoma [12,13]. Distinguishing PP from true progressive disease (PD) at the time of initial radiological progression remains challenging. Conventional response assessment is primarily based on the Response Evaluation Criteria in Solid Tumors (RECIST) [14], while the iRECIST criteria were introduced in 2017 to account for atypical response patterns associated with immunotherapy [15]. iRECIST introduced the concept of unconfirmed progressive disease (iUPD), requiring confirmation on subsequent imaging, generally performed 4–8 weeks later. However, neither conventional imaging nor response criteria can reliably distinguish PP from PD when progression is first observed. Clinicians may therefore face the difficult decision of continuing ICI pending reassessment, with the potential risk of maintaining ineffective treatment in patients with true progression [16].

This study aimed to characterize a real-world cohort of patients treated with ICIs in whom PP was clinically suspected and to explore routinely available clinical and biological factors associated with subsequent classification as PP versus PD.

## 2. Materials and Methods

### 2.1. Study Eligibility Criteria

We conducted a retrospective, single-center cohort study at the Institut de Cancérologie de l’Ouest (ICO). Eligible patients had metastatic cancer, irrespective of primary tumor site or histological subtype, were treated with immune checkpoint inhibitors (ICIs), and had clinically suspected pseudoprogression (PP) during follow-up.

The study was conducted in accordance with the Declaration of Helsinki and with authorization from the French National Commission for Information Technology and Civil Liberties (Commission Nationale de l’Informatique et des Libertés, CNIL). It was approved by the local independent ethics review board of Angers University Hospital (reference 2022-157). All surviving patients were informed of the study and provided written consent.

To identify potentially relevant cases, we searched the institutional medical-record database using the keywords “immunotherapy” and “pseudoprogression”. Records retrieved by this keyword-based search did not necessarily correspond to patients in whom PP had actually been considered a clinically plausible diagnosis; eligibility was subsequently determined by individual medical-record review according to the predefined study criteria. The initially identified records therefore did not represent all patients treated with ICIs at our institution. The total number of patients treated with ICIs during the study period was not collected, precluding estimation of the institutional incidence of PP. Patients with localized cancer, those receiving ICIs combined with chemotherapy or targeted therapy, those who discontinued ICI treatment before radiological reassessment because of death or toxicity, and those with incomplete medical records were excluded.

### 2.2. Data Collected

Clinical characteristics, treatment data, outcomes, and immune-related adverse events (irAEs) were retrospectively collected from medical records. IrAEs were graded according to the Common Terminology Criteria for Adverse Events (CTCAE), version 4.0. Performance status was assessed using the Eastern Cooperative Oncology Group (ECOG) scale.

Laboratory variables included hemoglobin, neutrophil count, albumin, calcium, and lactate dehydrogenase (LDH) levels. Clinical and biological data were collected at three predefined time points: ICI initiation, the time when PP was first clinically suspected (PP suspicion), and the subsequent evaluation at which patients were classified as PP or PD. Variables included in the logistic regression analysis were restricted to information available by the time of PP suspicion; no variable collected at the subsequent PP/PD classification was included in the model.

At the time of initial radiological progression during ICI treatment, PP could either be explicitly suggested in the radiology report or subsequently considered clinically plausible by the treating oncologist in the presence of discordance between radiological findings and the patient’s clinical condition. Radiological assessments were performed by radiologists as part of routine oncological care, in which RECIST 1.1 criteria and immune-related response patterns are routinely considered for patients receiving ICIs. No retrospective centralized radiological review was performed, and the systematic formal application of RECIST 1.1 or iRECIST criteria to all imaging examinations could therefore not be retrospectively verified. PP was considered clinically plausible when the treating physician decided to maintain ICI treatment despite initial radiological progression. Patients were subsequently classified as PP when follow-up imaging showed stabilization or reduction in the initially progressive disease without a change in systemic anticancer treatment, whereas continued radiological progression was classified as PD. The anatomical distribution of progression (primary tumor versus metastatic lesions) and mixed or dissociated response patterns were not systematically characterized on a lesion-by-lesion basis. No histopathological confirmation of suspected PP lesions was available; classification was therefore based on longitudinal clinical and radiological assessment.

The primary objective was to characterize patients with clinically suspected PP according to their subsequent classification as PP or PD. The secondary objective was to explore clinical and biological factors associated with subsequent classification as PP versus PD. A flowchart was constructed to describe the case-identification process, application of eligibility criteria, and subsequent classification of included patients as PP or PD.

### 2.3. Statistics

Descriptive statistics were used to summarize patient, disease, and treatment characteristics. Continuous variables are presented as medians and ranges, and categorical variables as numbers and percentages. Comparisons between patients subsequently classified as PP or PD were performed using non-parametric tests, as appropriate.

Overall survival (OS) was defined as the time from PP suspicion to death from any cause, with patients alive at last follow-up censored at that date, and was estimated using the Kaplan–Meier method. Median follow-up was estimated using the reverse Kaplan–Meier method from the date of ICI initiation.

Associations between candidate variables available by the time of PP suspicion and subsequent classification as PD versus PP were first assessed using univariable logistic regression. Variables showing an association with the outcome in univariable analysis (*p* < 0.05) were considered for the multivariable logistic regression model, together with clinically relevant potential confounders selected independently of statistical significance, namely primary tumor site, treatment line, and time from ICI initiation to PP suspicion. Results are reported as odds ratios (ORs) and adjusted odds ratios (aORs), with 95% confidence intervals (CIs). An OR > 1 indicates a higher probability of subsequent classification as PD. No automated stepwise variable-selection procedure was used. Given the limited effective sample size relative to the number of model parameters, the multivariable analysis was considered exploratory.

To explore the discriminative performance of LDH levels at the time of PP suspicion for distinguishing PD from PP, a receiver operating characteristic (ROC) curve was constructed and the area under the curve (AUC) with its 95% CI was estimated [17]. No data-derived LDH threshold was retained for clinical classification.

No formal sample-size calculation was performed because of the retrospective and exploratory nature of the study. All eligible patients identified during the predefined study period were included.

To assess the potential impact of missing data, characteristics of patients with and without missing weight and LDH values were compared. Because LDH missingness differed according to primary tumor site and the occurrence of immune-related adverse events (irAEs), sensitivity analyses were performed using deterministic median imputation stratified separately by these variables. The multivariable logistic regression model was then repeated using the imputed LDH values. All statistical tests were two-sided, and *p* < 0.05 was considered statistically significant. Statistical analyses were performed using R software (version 4.3.1), with the pROC, ROCR, and crosstable packages [18,19,20,21].

## 3. Results

**Figure 1:**
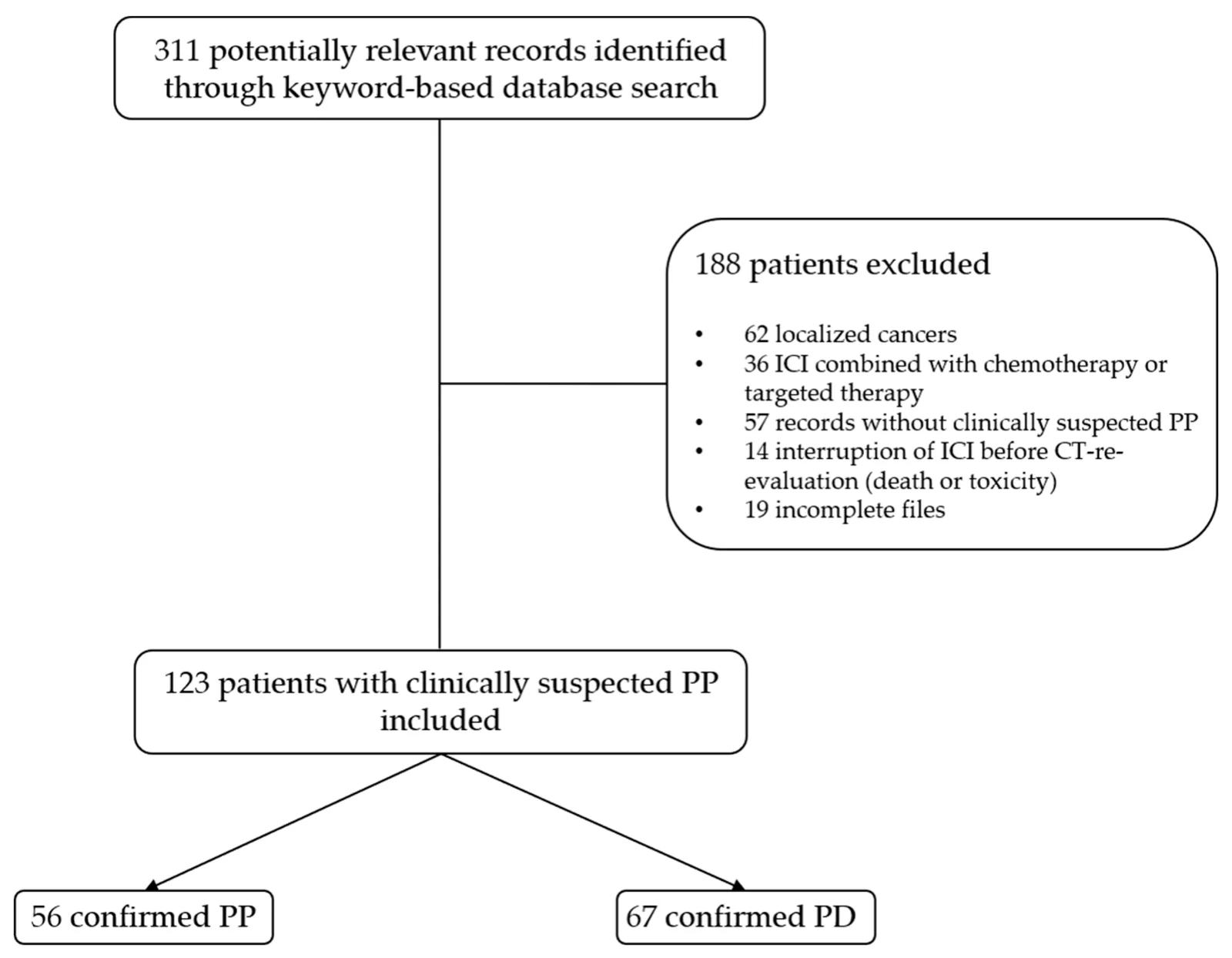
Flow chart.

From September 2013 to June 2022, the keyword-based database search identified 311 potentially relevant medical records in which the words “immunotherapy” and “pseudoprogression” were mentioned. These records did not represent the entire population of patients treated with ICIs at our institution during the study period. Individual medical-record review was subsequently performed to determine eligibility according to the predefined study criteria. Notably, 57 records had been retrieved because the word “pseudoprogression” appeared in the medical record, although PP had not actually been considered a clinically plausible diagnosis. These patients therefore did not meet the inclusion criterion of clinically suspected PP. After application of all eligibility criteria, 123 patients with clinically suspected PP were included in the analysis, of whom 56 were subsequently classified as PP and 67 as PD (Figure 1).

### 3.1. Characteristics at ICI Treatment Initiation

Overall, 59% of patients were male. Median age was 62 years (range, 13–81) at diagnosis and 65 years (range, 14–85) at ICI initiation. The most frequent primary tumor sites were lung (38%), kidney (24%), and bladder (11%), and patients had a median of two metastatic sites (range, 1–8). Baseline demographic, tumor, metastatic, and biological characteristics did not differ significantly between patients subsequently classified as PP and those classified as PD (Table 1 and Table S1).

ICIs were most commonly administered as second-line therapy, and anti-PD-1 monotherapy was the most frequently used regimen (84%). ICI type and treatment line did not differ significantly between the PP and PD groups. At ICI initiation, ECOG performance status differed between groups (*p* = 0.046), with ECOG status of 0 observed in 55% of patients subsequently classified as PP compared with 32% of those classified as PD (Table 1).

### 3.2. Characteristics at PP Suspicion

At the time of PP suspicion, all patients had radiological findings interpreted as disease progression on routine-care imaging assessment. PP was either explicitly suggested in the radiology report or subsequently considered clinically plausible by the treating oncologist based on the overall clinical context. ICI treatment was therefore continued pending radiological reassessment. PP was suspected at a median of 79 days (range, 28–927) after ICI initiation, most often at the first evaluation scan. Follow-up imaging was performed after a median of 66 days (range, 6–204) following PP suspicion (Table S2).

ECOG performance status did not differ significantly between patients subsequently classified as PP and those classified as PD. ECOG performance status was 0 in 36% versus 30%, 1 in 57% versus 64%, and 2 in 7% versus 6% of patients in the PP and PD groups, respectively. No patient had an ECOG performance status of 3 or 4 in either group at this time point (Table 1). There was no significant difference in weight or weight change from ICI initiation to PP suspicion between groups.

**Figure 2:**
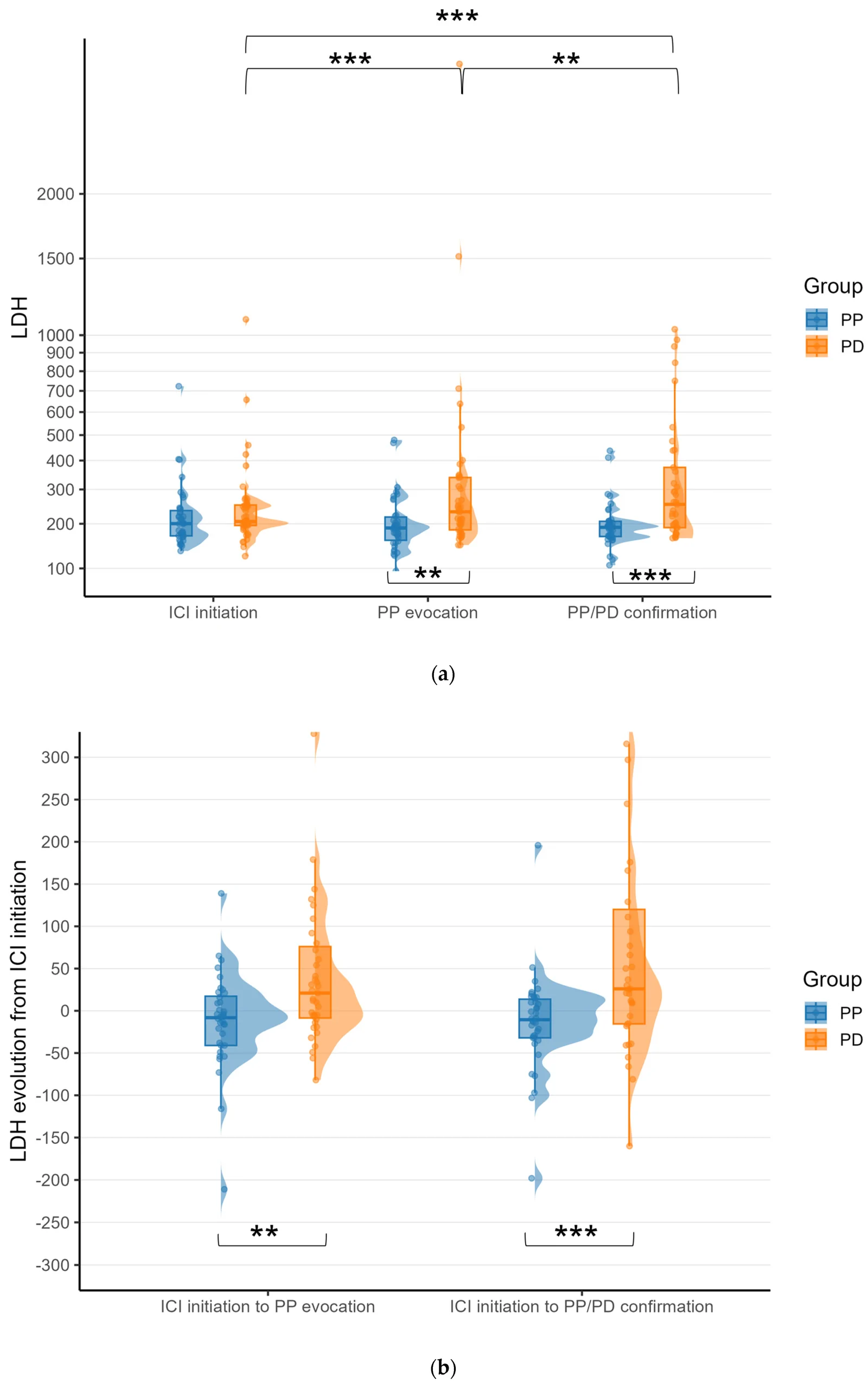
Boxplots representing levels (**a**) and variations in LDH (**b**) at the initiation of the immune checkpoint inhibitor (ICI) treatment, at the suspicion of pseudoprogression (PP), and at the final evaluation, at which PP (pseudoprogression group) or PD (progressive disease group) was classified. ** *p* < 0.01, *** *p* < 0.001.

At PP suspicion, no significant differences between the PP and PD groups were observed in albumin, hemoglobin, calcium, or neutrophil levels. Changes in albumin, hemoglobin, and calcium from ICI initiation to PP suspicion also did not differ significantly between groups. However, the change in neutrophil count over this period was greater in the PD group than in the PP group (*p* = 0.04) (Table S2 and Figure S1). LDH levels at PP suspicion were higher in patients subsequently classified as PD than in those classified as PP (median, 232.5 vs. 189 U/L; *p* = 0.003). From ICI initiation to PP suspicion, LDH levels increased in the PD group (median change, +8.5 U/L) but decreased in the PP group (median change, −4.2 U/L; *p* = 0.002) (Table S2 and Figure 2).

**Figure 3:**
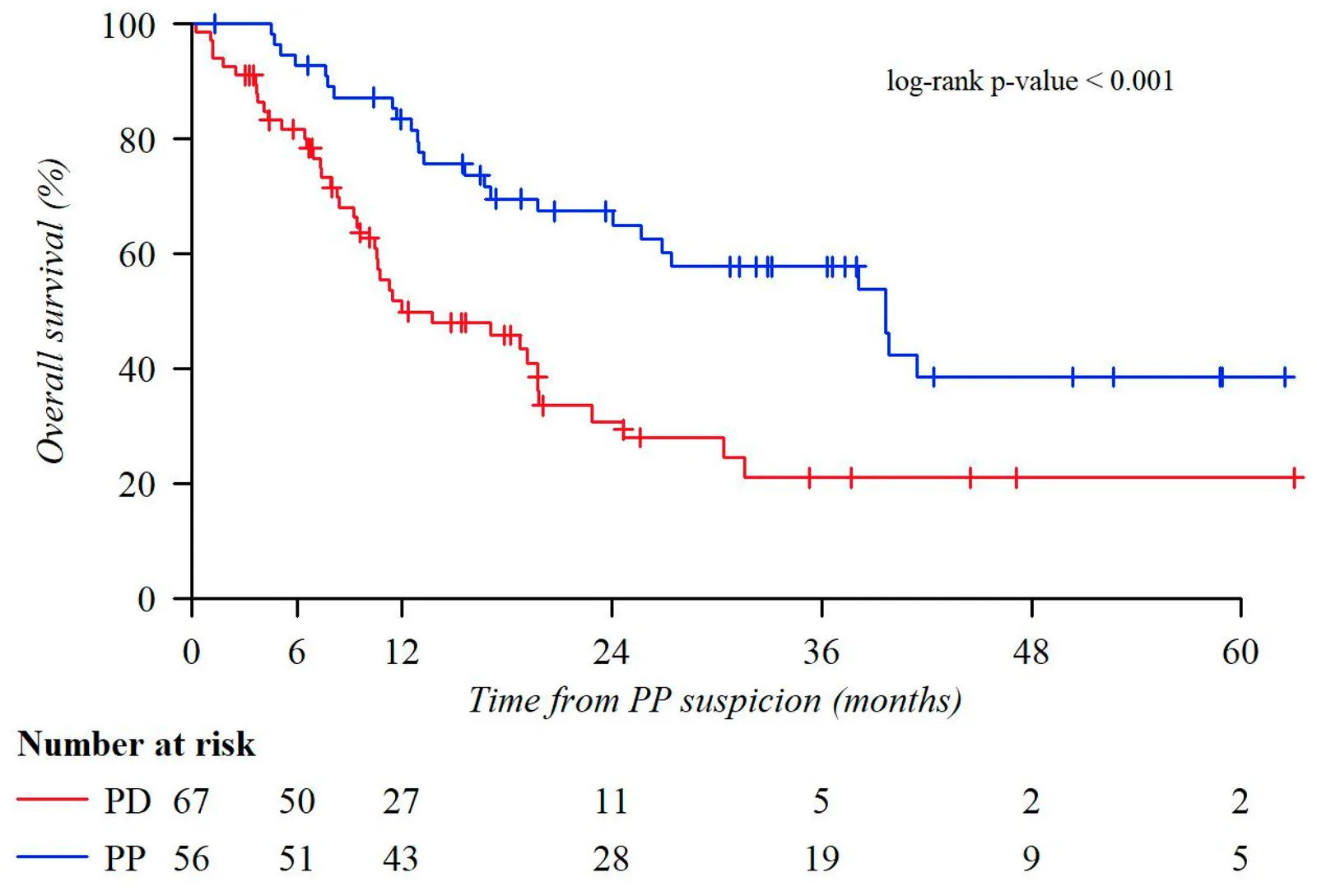
Kaplan–Meier survival curves for overall survival (OS) between the pseudoprogression (PP) group (blue curve) and the progressive disease (PD) group (red curve). The median OS was estimated to be 40 months in the PP group versus 12 months in the PD group (*p* < 0.001).

Median follow-up was 37.9 months (95% CI, 32.8–53.0), estimated using the reverse Kaplan–Meier method from ICI initiation. Median OS from PP suspicion was 39.7 months in the PP group compared with 12.0 months in the PD group (*p* < 0.001) (Figure 3).

### 3.3. Multivariable Analysis and Discriminative Performance of LDH

In univariable analysis, higher LDH levels at the time of PP suspicion were associated with a higher probability of subsequent classification as PD (OR 1.90 per 100 U/L increase; 95% CI, 1.07–3.37; *p* = 0.029). This association remained statistically significant after multivariable adjustment (aOR 2.78; 95% CI, 1.19–6.52; *p* = 0.019) (Table 2). The occurrence of ≥1 irAE during ICI treatment was associated with a lower probability of PD in univariable analysis (OR 0.46; 95% CI, 0.21–0.98; *p* = 0.049), but this association did not remain statistically significant after adjustment (aOR 0.30; 95% CI, 0.08–1.14; *p* = 0.08). None of the other variables included in the multivariable model, including time from ICI initiation to PP suspicion, change in neutrophil count, ECOG performance status at ICI initiation, primary tumor site, and treatment line, reached statistical significance after adjustment.

Missing LDH values were more frequent among patients with lung cancer and among those without irAEs, whereas no significant differences were identified according to missing weight status. Sensitivity analyses using median LDH imputation stratified by primary tumor site or occurrence of irAEs yielded adjusted estimates consistent with the complete-case analysis (aOR 2.82; 95% CI, 1.19–6.64; *p* = 0.018 and aOR 2.69; 95% CI, 1.18–6.09; *p* = 0.018, respectively). LDH levels at PP suspicion showed moderate discriminative performance for subsequent classification as PD, with an AUC of 0.70 (95% CI, 0.56–0.80) (Figure S2).

### 3.4. Immune-Related Adverse Events and Subsequent Treatment

At least one irAE occurred during ICI treatment in 41% of patients subsequently classified as PP and 24% of those classified as PD (*p* = 0.05). The most common irAEs were thyroid dysfunction, diarrhea, and skin disorders. IrAE severity did not differ significantly between groups; most events were grade 1–2 (Table 1).

Following classification as PP, 53 of 56 patients continued ICI treatment, whereas three discontinued treatment because of toxicity. Among the 53 patients who continued, ICI treatment was maintained in two despite uncertainty regarding the attribution of an adverse event to ICIs. In the PD group, subsequent treatment included chemotherapy in 44% and targeted therapy in 14%, while 18% discontinued systemic anticancer treatment. Ten patients continued ICI treatment beyond progression: two because progression was considered minimal or uncertain and eight in combination with local radiotherapy to a progressing metastatic site while the remaining disease sites were controlled.

### 3.5. Characteristics at Subsequent PP/PD Classification

At the subsequent evaluation, clinical deterioration was more frequent among patients classified as PD. Eleven patients in the PD group had deteriorated to an ECOG performance status of 3 or 4, whereas no patient in the PP group had an ECOG performance status of 3 or 4. Overall, 93% of patients in the PP group versus 59% in the PD group had an ECOG performance status of 0 or 1 (*p* < 0.0001). Weight loss was also more frequent in the PD group (*p* = 0.001) (Table S2).

At this time point, median hemoglobin levels were higher in the PP than in the PD group (13.2 vs. 12.1 g/dL; *p* = 0.014), whereas median LDH levels were lower (190 vs. 254 U/L; *p* < 0.0001). No significant differences were observed in albumin, neutrophil count, or calcium levels. Between PP suspicion and subsequent PP/PD classification, albumin and hemoglobin changes differed significantly between groups (*p* = 0.0125 and *p* = 0.0195, respectively), whereas changes in neutrophil count, calcium, and LDH did not (Table S2, Figure S1 and Figure 3).

## 4. Discussion

This retrospective pan-tumor study characterized patients treated with ICIs in whom PP was clinically suspected and explored routinely available clinical and biological features associated with subsequent classification as PP or PD. Importantly, our study was not designed to estimate the incidence of PP among patients receiving ICIs. Case identification relied on a keyword-based search specifically targeting medical records in which PP had been mentioned, and the total population of patients treated with ICIs during the study period was not available. Accordingly, the distribution of PP and PD observed in this selected cohort should not be interpreted as reflecting the incidence or probability of PP in the overall ICI-treated population.

PP was observed across a broad range of malignancies in our selected cohort, including lung, renal, urothelial, and melanoma. Previous studies have reported varying rates of PP according to tumor type [22,23], although our study was not designed to assess tumor-specific differences. The median time from ICI initiation to PP suspicion was 79 days, most often corresponding to the first imaging assessment. This is consistent with previous reports showing that PP generally occurs relatively early after ICI initiation, although delayed cases have also been described [12,24,25]. Recognition of these atypical response patterns remains particularly relevant both in routine clinical practice and in the design and interpretation of immunotherapy trials [26].

Patients subsequently classified as PP had substantially longer OS than those classified as PD, consistent with previous reports [24]. This finding should nevertheless be interpreted descriptively, as survival was not the primary endpoint and the analysis was not adjusted for differences in subsequent treatment strategies or residual confounding. Histopathological observations also support a biological basis for PP. In a systematic review of PP in non-small cell lung cancer, Chen et al. reported that biopsies of suspected PP lesions frequently showed inflammatory changes, with infiltrates predominantly composed of CD4+ and CD8+ T cells and macrophages [22]. More broadly, the distinction between PP and true progression occurs within the complex biological landscape of response and resistance to ICIs. Primary and acquired resistance may involve multiple tumor-intrinsic and immune-mediated mechanisms, including impaired antigen presentation, T-cell dysfunction and exhaustion, an immunosuppressive tumor microenvironment, and regulatory mechanisms involving non-coding RNAs such as microRNAs [7,8]. This biological complexity further supports a multidimensional assessment of suspected PP rather than reliance on a single clinical or biological parameter.

Among routinely available clinical parameters, baseline performance status differed between groups, with ECOG performance status of 0 being more frequent among patients subsequently classified as PP. This observation is consistent with previous reports suggesting that maintenance of a good general condition may support the clinical suspicion of PP [27,28,29]. In the subgroup analysis of the phase III CheckMate 025 trial, patients with advanced renal cell carcinoma who continued nivolumab beyond RECIST-defined progression and subsequently experienced tumor reduction more frequently had a preserved performance status [23]. However, ECOG performance status at the time of PP suspicion did not significantly differ between groups in our cohort. Clinical condition should therefore be considered as part of the overall assessment rather than as an isolated discriminator between PP and PD. Our secondary objective was to explore routinely available clinical and biological features associated with subsequent confirmation of PP or PD.

Some studies have also suggested an association between immune-related adverse events and PP [30]. In our cohort, the occurrence of at least one irAE during ICI treatment was associated with a lower probability of PD in univariable analysis (OR 0.46; 95% CI, 0.21–0.98; *p* = 0.049), but this association did not remain statistically significant after multivariable adjustment (aOR 0.30; 95% CI, 0.08–1.14; *p* = 0.08). This finding should therefore be interpreted cautiously. Moreover, irAEs were analyzed as a binary variable, without accounting for their type, grade, timing, or multiplicity, which may have oversimplified their potential relationship with PP.

Among the biological variables evaluated, LDH emerged as the most consistent factor associated with subsequent classification. Higher LDH levels at the time of PP suspicion remained associated with PD after multivariable adjustment. This observation is consistent with previous studies suggesting that LDH reflects tumor metabolic activity and overall disease burden. Kim et al. reported an association between elevated baseline LDH and hyperprogressive disease under ICIs, whereas Basler et al. found lower LDH levels to be independently associated with PP in metastatic melanoma [31,32]. However, the discriminative performance of LDH in our cohort was only moderate (AUC 0.70), indicating that LDH alone cannot reliably distinguish PP from PD. Furthermore, absolute LDH levels may be influenced by tumor type and disease burden, and the absence of standardized normalization to laboratory-specific upper limits of normal further limits the generalizability of this association in our pan-tumor cohort. Consistently, Basler et al. reported an AUC of 0.71 for a model combining biological parameters including LDH and S100, which increased to 0.79 when radiomic and biological features were combined [32]. These findings support the integration of LDH with other clinical or imaging parameters rather than its use as a standalone biomarker.

More comprehensive approaches may therefore improve the distinction between PP and PD. Radiomics offers the possibility of extracting quantitative information related to tumor morphology and heterogeneity from routinely acquired imaging [33,34]. In a cohort of 33 patients with non-small cell lung cancer treated with ICIs, Barabino et al. reported radiomic features associated with PD [35]. Circulating tumor DNA (ctDNA) may provide another complementary approach [36]. Guibert et al. reported decreasing KRAS-mutated ctDNA in patients experiencing PP, whereas persistently elevated levels were observed in a patient with PD [37]. Similarly, Lee et al. showed that ctDNA kinetics could discriminate PP from PD in patients with melanoma treated with ICI [38]. Although these approaches require further prospective validation, they illustrate the potential value of combining clinical, biological, and imaging information when PP is suspected.

Taken together, our findings indicate that no routinely available clinical or biological parameter evaluated in this study can independently and reliably distinguish PP from PD. In clinically stable patients with suspected PP, confirmatory imaging therefore remains essential, while clinical condition, irAEs, and LDH may provide complementary information during this period of diagnostic uncertainty.

Several limitations should be acknowledged. First, the retrospective keyword-based case-identification strategy introduced an inherent selection bias, as only patients in whom PP was explicitly mentioned in the medical record could be identified. Patients in whom PP was not considered or documented were therefore not captured, precluding estimation of PP incidence and limiting generalizability to the broader ICI-treated population. Second, the sample size was limited for multivariable and subgroup analyses, particularly given the pan-tumor design and the number of model parameters. The adjusted analyses should therefore be considered exploratory rather than constituting a validated predictive model, and tumor-specific or treatment-specific conclusions cannot be drawn. Third, missing data affected several variables, particularly LDH, and missingness was associated with primary tumor site and occurrence of irAEs. Although sensitivity analyses using stratified median imputation yielded results consistent with the complete-case analysis, residual bias cannot be excluded. The retrospective radiological assessment represents another important limitation. No centralized retrospective radiological review was performed, and although RECIST criteria and immune-related response patterns were considered as part of routine oncological care, systematic formal application of RECIST 1.1 or iRECIST to every imaging examination could not be retrospectively verified. The anatomical distribution of progression and mixed or dissociated response patterns were not systematically characterized on a lesion-by-lesion basis. In addition, the interval between initial PP suspicion and follow-up imaging varied substantially (median, 66 days; range, 6–204), reflecting the absence of a protocol-defined reassessment schedule. Finally, no histopathological confirmation of suspected PP lesions was available. Classification therefore relied on longitudinal clinical and radiological assessment, and some cases classified as PP may have represented delayed responses or other atypical response kinetics. These limitations emphasize the need for prospective studies incorporating standardized imaging schedules, centralized response assessment, and multimodal biomarkers in larger, preferably tumor-specific cohorts.

## 5. Conclusions

Pseudoprogression represents an important diagnostic challenge in patients treated with ICIs. In this real-world pan-tumor cohort of patients with clinically suspected PP, higher LDH levels at the time of PP suspicion were associated with subsequent classification as PD, but their discriminative performance was insufficient to reliably distinguish PP from PD when considered alone. No routinely available clinical or biological parameter identified in our study could independently establish the diagnosis of PP. Confirmatory imaging therefore remains essential, while clinical condition and routinely available biological parameters such as LDH may provide complementary information during this period of diagnostic uncertainty. Prospective studies incorporating standardized imaging assessment and multimodal biomarkers are needed to validate these findings and improve the distinction between PP and true progression.

## Figures and Tables

**Table 1:** Characteristics at initiation of immune checkpoint inhibitor treatment and at PP suspicion.

Baseline Characteristics		Pseudoprogression N = 56	Progressive Disease N = 67	*p*-Value
**Gender**	Male	31 (55%)	42 (63%)	0.41
	Female	25 (45%)	25 (37%)	
**Age at diagnosis**	Median (range)	64 (13–81)	60 (33–77)	0.20
**Primary site**	Skin	5 (8%)	4 (6%)	0.47
	Digestive	2 (4%)	1 (2%)	
	Gynecologic	1 (2%)	4 (6%)	
	ORL	8 (14%)	5 (8%)	
	Lung	20 (36%)	27 (40%)	
	Kidney	16 (29%)	14 (21%)	
	Breast	-	2 (3%)	
	Central Nervous System	-	1 (2%)	
	Bladder	4 (7%)	9 (13%)	
**Histological type**	Adenocarcinoma	19 (34%)	28 (42%)	0.81
	Squamous cell carcinoma	11 (20%)	10 (15%)	
	Undifferentiated carcinoma	1 (2%)	1 (2%)	
	High-grade salivary carcinoma	1 (2%)	-	
	Urothelial carcinoma	6 (11%)	9 (13%)	
	Infiltrating ductal carcinoma	-	2 (3%)	
	Clear cell renal carcinoma	14 (25%)	13 (19%)	
	melanoma	4 (7%)	4 (6%)	
**Age at ICI initiation**	Median (range)	66.5 (14–82)	64 (38–85)	0.23
**Weight at ICI initiation ¥**	Median (range)	73 (40–123)	70.5 (37–108)	
ECOG * at ICI initiation	0	30 (55%)	21 (32%)	**0.046**
	1	21 (38%)	38 (59%)	
	2	4 (7%)	6 (9%)	
**Type of ICI**	Anti-PD-1	48 (86%)	56 (84%)	0.99
	Anti-PD-1 + Anti-PD-L1	-	1 (1%)	
	Anti-PD-L1	5 (9%)	6 (9%)	
	Anti-PD-1 + Anti-CTLA-4	3 (5%)	4 (6%)	
**Line of ICI use**	Median (range)	2 (1–5)	2 (1–8)	0.56
**Number of metastatic sites**	Median (range)	2 (1–6)	2 (1–8)	0.16
**Metastatic sites at ICI initiation**	Bone	10 (18%)	18 (27%)	0.24
	Liver	12 (21%)	19 (28%)	0.38
	Lymph nodes	28 (50%)	42 (63%)	0.16
	Lung	34 (61%)	41 (61%)	0.96
	Brain	7 (13%)	13 (19%)	0.30
	Skin	2 (4%)	2 (3%)	0.99
	Adrenal	7 (13%)	9 (13%)	0.88
**Time between ICI initiation and PP suspicion**	Median (range)	79 (28–227)	78 (28–927)	0.91
ECOG ** at PP suspicion	0	20 (36%)	20 (30%)	
	1	32 (57%)	42 (64%)	
	2	4 (7%)	4 (6%)	
Immune-related adverse event *	At least one	23 (41%)	16 (24%)	**0.05**
	No one	33 (59%)	50 (75%)	
	Grade 1/2	19 (34%)	16 (24%)	0.22
	Grade 3/4	5 (9%)	2 (3%)	0.25
**Metastatic site in progression at PP suspicion**	Bone	12 (21%)	17 (25%)	0.24
	Liver	23 (41%)	38 (57%)	0.38
	Lymph node	29 (52%)	34 (51%)	0.16
	Lung	3 (5%)	1 (2%)	0.96
	Brain	2 (4%)	2 (3%)	0.30
	Skin	2 (4%)	3 (5%)	0.99
	Adrenal	12 (21%)	17 (25%)	0.88

¥: 5 patients had missing values (4 in PP group and 1 in PD group). *: 3 patients had missing values (2 in the PP group and 1 in the PD group). **: 1 patient had a missing value (in the PP group). Abbreviations: ICI, immune checkpoint inhibitor; ECOG, Eastern Cooperative Oncology Group; *PD-1, programmed cell death 1*; *PD-L1, programmed death-ligand 1*; *CTLA-4, cytotoxic T-lymphocyte-associated antigen 4*.

**Table 2:** Univariable and multivariable logistic regression analyses of factors associated with subsequent classification as PD versus PP.

	Univariable Model		Multivariable Model	
Characteristic	OR (95% CI)	*p*-Value	Adjusted OR (95% CI)	*p*-Value
Time from ICI initiation to PP suspicion (one month more)	1.17 (0.92 to 1.49)	0.20	1.37 (0.81 to 2.3)	0.24
Neutrophil count variation between ICI initiation and PP suspicion (1000 units more)	1.29 (1.04 to 1.59)	**0.021**	1.38 (0.96 to 1.99)	0.08
LDH at PP suspicion time (100 units more)	1.90 (1.07 to 3.37)	**0.029**	2.78 (1.19 to 6.52)	**0.019**
Occurrence of ≥1 immune-related adverse event during ICI treatment	0.46 (0.21 to 0.98)	**0.049**	0.3 (0.08 to 1.14)	0.08
ECOG at ICI initiation				
ECOG 0	1		1	
ECOG 1	2.59 (1.2 to 5.59)	**0.016**	2.03 (0.62 to 6.59)	0.24
ECOG 2	2.14 (0.54 to 8.54)	0.28	6.37 (0.54 to 75.08)	0.14
Primary tumor site				
Lung	1		1	
Renal	0.65 (0.26 to 1.63)	0.36	1.14 (0.24 to 5.48)	0.87
Other primary tumor sites	0.96 (0.42 to 2.19)	0.93	0.91 (0.22 to 3.82)	0.90
Treatment Line				
1st line	1		1	
Second line	1.08 (0.45 to 2.6)	0.86	0.45 (0.11 to 1.82)	0.26
Third or later line	1.46 (0.43 to 4.9)	0.54	0.91 (0.14 to 5.94)	0.92

Abbreviations: CI, confidence interval; OR, odds ratio; ECOG, Eastern Cooperative Oncology Group; ICI, immune checkpoint inhibitor; LDH, lactate dehydrogenase.

## Data Availability

The original contributions presented in this study are included in the article/Supplementary Materials. Further inquiries can be directed to the corresponding author.

## Supplementary Materials

The following supporting information can be downloaded at: https://www.mdpi.com/article/10.3390/cancers18183040/s1, Figure S1. Boxplots representing levels and variations in albumin (A), hemoglobin (B), neutrophils (C), and calcium (D) at ICI initiation, at the suspicion of PP, and at the final evaluation when PP (pseudoprogression group) or PD (progressive disease group) was classified. * *p* < 0.05, ** *p* < 0.01, *** *p* < 0.001; Figure S2. ROC curves according to LDH levels (black curve) and variations in the LDH levels between ICI treatment initiation and pseudoprogression (PP) suspicion (red curve). Table S1. Baseline biological characteristics; Table S2. Characteristics of the patient population at the final evaluation at which PP or PD was classified.

## Abbreviations

The following abbreviations are used in this manuscript:

ICI	Immune checkpoint inhibitor
PD	Progressive disease
PP	Pseudoprogression
ICO	Institut de Cancérologie de l’Ouest
CI	Confidence intervals
CTLA-4	Cytotoxic T-lymphocyte-associated antigen 4
PD-1	Programmed cell death protein 1
PD-L1	Programmed death-ligand 1
RECIST	Response Evaluation Criteria in Solid Tumors
iUPD	Unconfirmed progressive disease
TRAEs	Treatment-related adverse events
ECOG	Eastern Cooperative Oncology Group
ORL	Head and neck cancer
LDH	Lactate dehydrogenase
CT	Evaluation tomography
OS	Overall survival
OR	Odds ratio
ROC	Receiver operating curve
AUC	Area under the curve
