# Supplementary material for: Clinical and Biological Factors Associated with Pseudoprogression Under Immune Checkpoint Inhibitors: A Retrospective Pan-Tumor Cohort Study
Source: Cancers (Basel). 2026 Sep 19;18(18):3040. doi: 10.3390/cancers18183040 (PMC13604733; doi:10.3390/cancers18183040)
Supplement: Supplementary file 1 [file cancers-18-03040-s001.zip › cancers-4505477-supplementary.pdf]

Supplementary Tables

Table S1. Baseline biological characteristics.

Baseline Characteristics		Overall Cohort N = 123	Pseudoprogression N = 56	Progressive Disease N = 67	*p*-Value
**Albumin**	median (range)	40 (25.2–46.8)	40 (26–46.8)	40.4 (25.2–46)	0.78
	Na	n = 53	n = 27	n = 26	
**Hemoglobin**	median (range)	12.5 (8.1–16.1)	12.5 (8.5–15.6)	12.4 (8.1–16.1)	0.47
	N	n = 17	n = 7	n = 10	
**Neutrophils**	median (range)	4460 (1410–16,200)	4554.5 (2090–16,200)	3843 (1410–15,790)	0.21
	Na	n = 20	n = 8	n = 12	
**LDH**	median (range)	205 (124–1095)	200.2 (135–723)	206 (124–1095)	0.13
	Na	n = 37	n = 17	n = 20	
**Calcemia**	median (range)	2.4 (2.1–2.9)	2.4 (2.1–2.7)	2.4 (2.2–2.9)	0.60
	Na	n = 52	n = 26	n = 26	

Abbreviations: LDH, lactate dehydrogenase; Na, unknown.

Table S2. Characteristics of the patient population at the final evaluation at which PP or PD was classified.

Characteristics at the Final Evaluation		Overall Cohort N = 123	Pseudoprogression N = 56	Progressive Disease N = 67
**ECOG *****	0	29 (25%)	16 (29%)	13 (21%)
	1	59 (50%)	35 (64%)	24 (38%)
	2	19 (16%)	4 (7%)	15 (24%)
	3	9 (7%)	-	9 (14%)
	4	2 (2%)	-	2 (3%)
**Time between PP suspicion and first scan reevaluation** (days)	median (range)	66 (47–86)	63 (49–86)	66 (42–86)
	Na	n = 2	n = 1	n = 1
**Weight**	median (range)	69.5 (38–117)	67.5 (38–117)	70.5 (38–111)
	Na	n = 11	n = 6	n = 5
**Relative weight variation from ICI treatment initiation *****	median (range)	−0.9 (−17.4 + 14.9)	0 (−14.7–15)	−1.6 (−15.3–14.9)
	Na	n = 13	n = 6	n = 7
**Albumin**	median (range)	39 (16–47.5)	41 (16–47.5)	38 (19–46.7)
	Na	n = 52	n = 25	n = 27
**Relative albumin variation from ICI treatment initiation ***	median (range)	0.0 (−28.1 + 19.4)	2.5 (−5.4 + 19.4)	−0.7 (−28 + 12.1)
	Na	n = 63	n = 29	n = 34
**Hemoglobin ***	median (range)	12.9 (7.1–17)	13.2 (9.1–16.6)	12.1 (7.1–17)
	Na	n = 21	n = 10	n = 11
**Relative hemoglobin variation from ICI treatment initiation ***	median (range)	0.7 (−35.7 + 22.1)	1.6 (−7.6 + 22.1)	−1.5 (−35.7 + 20.5)
	Na	n = 25	n = 11	n = 14
**Neutrophils**	median (range)	4799 (1811–18,290)	4671 (2097–18,170)	5480 (1811–18,290)
	Na	n = 22	n = 11	n = 11
**Relative neutrophil variation from ICI treatment initiation**	median (range)	9.4 (−44.9 + 206.9)	3.0 (−43.7 + 185.8)	11.1 (−44.9 + 206.9)
	Na	n = 27	n = 12	n = 15
**LDH *****	median (range)	198 (106–1035)	190.8 (106–437)	254 (164–1035)
	Na	n = 46	n = 16	n = 30
**Relative LDH variation from**	median (range)	5.6 (−71.0 + 93.8)	−2.6 (−50.4 + 51.6)	8.0 (−71 + 93.8)

**ICI treatment initiation**				
	Na	n = 51	n = 19	n = 32
**Calcemia**	median (range)	2.4 (2.2–2.8)	2.4 (2.2–2.6)	2.4 (2.2–2.8)
	Na	n = 52	n = 23	n = 29
**Relative calcemia variation from ICI treatment initiation**	median (range)	0.0 (−14.0 + 25.7)	0 (−10.6 + 25.7)	0.2 (−14.0 + 11.5)
	Na	n = 59	n = 26	n = 33

Abbreviations: ECOG, Eastern Cooperative Oncology Group; ICI, immune checkpoint inhibitor; PP, pseudo-progression; LDH, lactate dehydrogenase; Na, unknown. p-Values were obtained using the two-sided Mann–Whitney test (p < 0.05; *** p < 0.001).*

Supplementary Figures

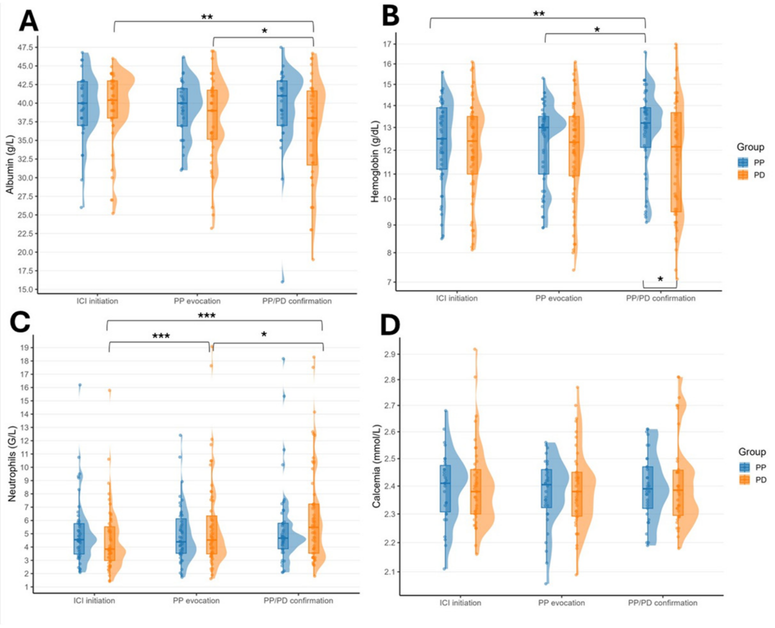

Figure S1. Boxplots representing levels and variations in albumin (**A**), hemoglobin (**B**), neutrophils (**C**), and calcium (**D**) at ICI initiation, at the suspicion of PP, and at the final evaluation when PP (pseudoprogression group) or PD (progressive disease group) was classified. * $p < 0.05$, ** $p < 0.01$, *** $p < 0.001$.

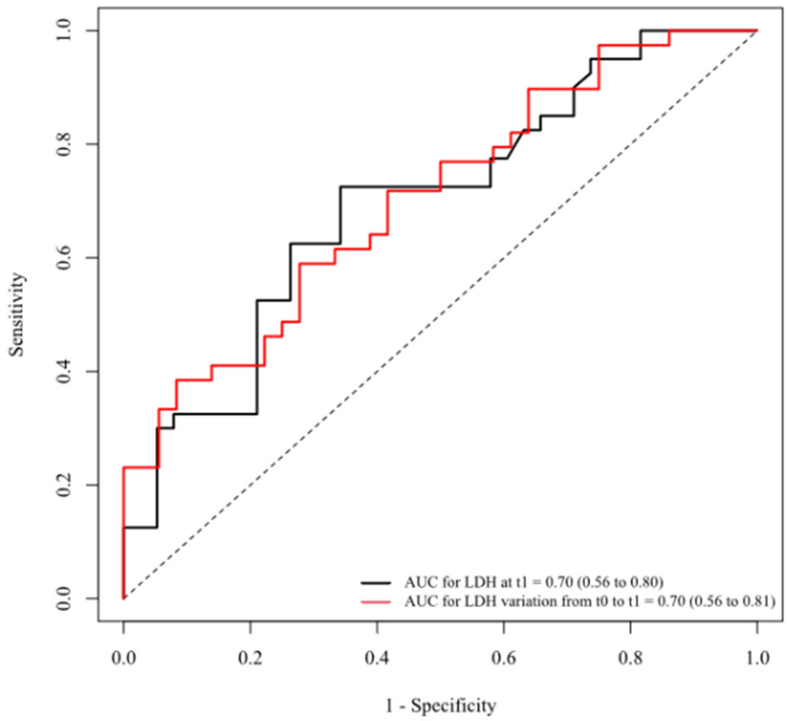

Figure S2. ROC curves according to LDH levels (black curve) and variations in the LDH levels between ICI treatment initiation and pseudoprogression (PP) suspicion (red curve).
